# Highly Porous Tantalum Acetabular Components Without Ancillary Screws Are Non-inferior at 7 Years When Compared With Titanium Components With Ancillary Screw Fixation: A Randomized Controlled Trial

**DOI:** 10.1016/j.artd.2025.101709

**Published:** 2025-05-17

**Authors:** Thomas S. Robertson, Lucian B. Solomon, Donald W. Howie, Oksana T. Holubowycz, Chan Hee Cho, Stuart A. Callary

**Affiliations:** a Department of Orthopaedics and Trauma, Royal Adelaide Hospital, Adelaide, South Australia, Australia; b Centre for Orthopaedic and Trauma Research, Faculty of Health and Medical Sciences, The University of Adelaide, Adelaide, South Australia, Australia

**Keywords:** RSA, Tantalum, THR

## Abstract

**Background:**

While porous tantalum components have shown to be advantageous in the revision setting, registry studies have identified tantalum components used in primary total hip arthroplasty (THA) to be associated with an increased risk of revision. The only study to examine the migration of tantalum acetabular components with radiostereometric analysis (RSA) beyond 2 years found continued migration. The aim of this 7-year follow-up RSA study was to determine if the mid-term migration of tantalum acetabular components without ancillary screw fixation is no greater than that of fiber metal titanium components with one ancillary screw fixation.

**Methods:**

We prospectively reviewed the mid-term implant stability of patients who underwent primary THA and were randomized intra-operatively to receive either the tantalum or titanium acetabular component. Of the initial 66 patients enrolled, 51 (77.3%) were available at 7-year follow-up; 2 tantalum components were revised due to recurrent dislocation and infection, respectively, and 2 titanium components underwent open reduction internal fixation to treat femoral periprosthetic fracture. Acetabular component migration relative to the surrounding acetabular bone was measured using RSA at 4-6 days post-operatively and at 6 weeks, 3 months, 1, 2, 3, 5, and 7 years following THA.

**Results:**

At 7 years, the mean proximal migration of tantalum components was 0.22 mm (95% confidence interval 0.08-0.35) and non-inferior to that of titanium components at 0.19 mm (95% confidence interval 0.07-0.32). In addition, the mean proximal migration of tantalum components was non-inferior to that of titanium at both 3 and 5 years. There were no significant differences noted between cohorts for any other axis of translation and rotation.

**Conclusions:**

The continued mid-term stability of tantalum acetabular components without ancillary screw fixation is encouraging for long-term stability. The non-inferiority compared to titanium acetabular components with established excellent long-term survivorship provides reassurance to the operative surgeon using tantalum components in the primary setting.

**Level of Evidence:**

I.

## Introduction

Porous tantalum acetabular components have been utilized to good success in revision total hip arthroplasty (THA); however, their success as a reliable primary component is yet unclear. The highly porous three-dimensional (3D) structure of the tantalum surface is believed to provide an ideal surface for biological in-growth; it also has low stiffness which reduces stress shielding [1,2]. These advantageous properties of tantalum include a higher co-efficient of friction than fibre-mesh titanium [3], which may provide adequate initial stability without the additional use of screws [3].

Two recent studies, including large-scale registry data, have identified tantalum acetabular components as being at risk of higher-than-anticipated revision rates by all causes overall [4,5]. Laaksonen et al. [4] demonstrated that in a registry study of 95,709 components even when accounting for all other confounding variables, tantalum cups had a persistently higher rate of revision with up to 8 years, with a survivorship of 94.4% compared to 96.2%. Despite published recommendations for caution on the use of tantalum components, it continues to be used for primary THA, as such further studies are required to ensure the safety of such use [6].

Early migration of acetabular components is known to be a predictor of long-term aseptic loosening requiring revision [[7], [8], [9]]. Radiostereometric analysis (RSA) is the most accurate form of radiographic imaging to measure the 3D micromotion of implants in vivo [10]. As such RSA studies have strong merit in the phased introduction of implants [11]. Migration of tantalum components at 2 years was reported as similar to titanium in our earlier report of this cohort [12] and 2 previous RSA studies [13,14]. However, continuing migration was reported for tantalum components between 22 and 5 years [14], which is theorized to predict failure by loosening [15].

The paucity of RSA migration data beyond 2 years, combined with registry evidence of increased revision rate of tantalum acetabular components used in primary THA, highlights the need for longer follow-up studies of this implant. The aim of this study was to determine if the mid-term migration of porous tantalum components was non-inferior to that of titanium components.

## Material and methods

The methods of this trial and RSA measurement are consistent and identical as the published 2-year results, and a more comprehensive methodology is available in that report [12]. The results of this trial are reported in accordance with CONSORT (Consolidated Standards of Reporting Trials) 2010 Guidelines and in line with recent RSA guidelines [16,17]. The study is a stratified, parallel-group randomized controlled trial (RCT) undertaken at the Royal Adelaide Hospital, a teaching and tertiary-care referral hospital. All procedures were performed by one of six attending surgeons or residents or fellows under their supervision. All patients in this trial were aged between 40 and 64 years at the time of primary THA, had a diagnosis of primary or secondary osteoarthritis, and reported that their walking was restricted only by their hips. Patients were randomized intra-operatively to receive either a cementless press-fit solid-backed tantalum acetabular component with no ancillary screw fixation or a cementless press-fit titanium fibre-metal acetabular component with ancillary screw fixation. Ethics approval was received from the hospital's institutional review board. This trial is registered with the Australian New Zealand Clinical Trials Registry (ACTRN12613000882729).

All patients who were to undergo primary THA by one of the participating surgeons were screened for inclusion in the trial. The reasons for and numbers of pre-operative exclusions are detailed in Appendix I. The randomization envelope was opened in the operating room after the acetabular trial had been inserted and was determined to fit firmly. The patient received either a titanium acetabular component 2 mm larger in diameter than the trial or a tantalum component of the same diameter as the trial (oversized 2 mm at the peripheral rim) according to the allocation in the envelope.

All arthroplasties were undertaken using a cemented polished femoral stem with a 12/14 taper (CPT; Zimmer, Warsaw, IN), a cobalt chromium 28-mm femoral head (Versys; Zimmer), and an uncemented modular acetabular component comprising a 10° elevated highly cross-linked polyethylene liner (Longevity; Zimmer) in either a cementless press-fit solid-backed tantalum acetabular shell (Trabecular Metal; Zimmer) with no screw fixation or a cementless press-fit cluster-holed titanium fibre-metal acetabular shell fixed with one screw (Trilogy; Zimmer).

Nine tantalum beads (1.0-mm diameter, RSA Biomedical, Umea, Sweden) were inserted intra-operatively into the periprosthetic acetabular bone of each patient. RSA radiographs were taken 4-6 days following THA and at 6 weeks, 3 months, 1 year, and 2 years after surgery, with the patient supine. A uniplanar RSA setup was used. Two radiographic tubes, a room-mounted unit (Philips Bucky Diagnost; Philips Healthcare, Andover, MA) and a mobile radiographic unit (Philips Practix 8000; Philips Healthcare), were positioned 1.6 m above a calibration cage (Cage no.43; RSA Biomedical) with a 40° angle between the tubes. The calibration cage contained 2 35 × 43-cm high-resolution digital radiographic cassettes. The radiographic tubes were exposed simultaneously at 120 kV and 16 mAs. Cassettes were digitized with an AGFA Centricity CR SP1001 processor (AGFA Healthcare, Mortsel, Belgium). Radiographs were analyzed using RSA software (UmRSA version 6.0 and UmRSA DICOM link; RSA Biomedical) by one of the authors (SC). The RSA radiographs at 4-6 days following THA were used as a baseline, and migration was calculated as the difference from the baseline at each time interval. Translation and rotation of the acetabular component in each axis were measured using the center of the acetabular component identified by the ellipse algorithm (unbeaded) [18] with reference to beads in the periprosthetic bone. Two-dimensional (2D) and 3D migration was calculated as the vectorial sum of translation in the coronal plane and all 3 axes, respectively. The results were analyzed and reported in accordance with suggested guidelines for reporting RSA studies [19]. The mean precision of proximal migration has been previously reported and noted to be low at 0.009 mm (99% confidence interval [CI] 0.010-0.029 mm) [12].

### Statistical analysis

Proximal migration at 5 and 7 years following THA was calculated for each individual. The aim of this study was to determine whether proximal migration of the tantalum components was no greater than the migration of the titanium components. Therefore, a one-sided test of non-inferiority was considered the most appropriate statistical test. The rationale for selecting −0.21 mm as the non-inferiority margin was based on the finding of a systematic review by Pijls et al. [7], which showed that a pooled mean proximal migration greater than 0.4 mm in 3 prosthesis designs was associated with a clinically unacceptable revision rate exceeding 5.7% at 10 years. In our previously reported results, the mean proximal migration of the titanium components in our study was 0.19 mm; hence, an additional migration of 0.21 mm would result in a mean migration of 0.4 mm. Therefore, proximal migration of the tantalum component was determined to be not inferior (ie, not greater) than that of the titanium component if the 95% (ie, 100 - 2∝%, where ∝ = 0.025) lower confidence limit of the difference between means did not exceed the lower bound of −0.21. For the 5-year analysis, there is no clear threshold presented in the literature for midterm follow-up of RSA. As such, the same threshold has again been utilized. To assess non-inferiority of the 2 treatments (tantalum vs titanium), we computed the two-sided 95% CI of the difference between treatments. Using this method, the experimental treatment (tantalum) is not inferior to the control treatment (titanium) at a 2.5% level if the lower boundary is above a pre-specified margin of non-inferiority; in this case, consider a non-inferiority limit of 0.21. Because the upper bound of the *t*-test is 0.199 or less than the non-inferiority limit, the test shows non-inferiority. Additional *P* values are presented using an unpaired *t*-test where equal variances are assumed for all parameters of RSA.

## Results

**Figure 1:**
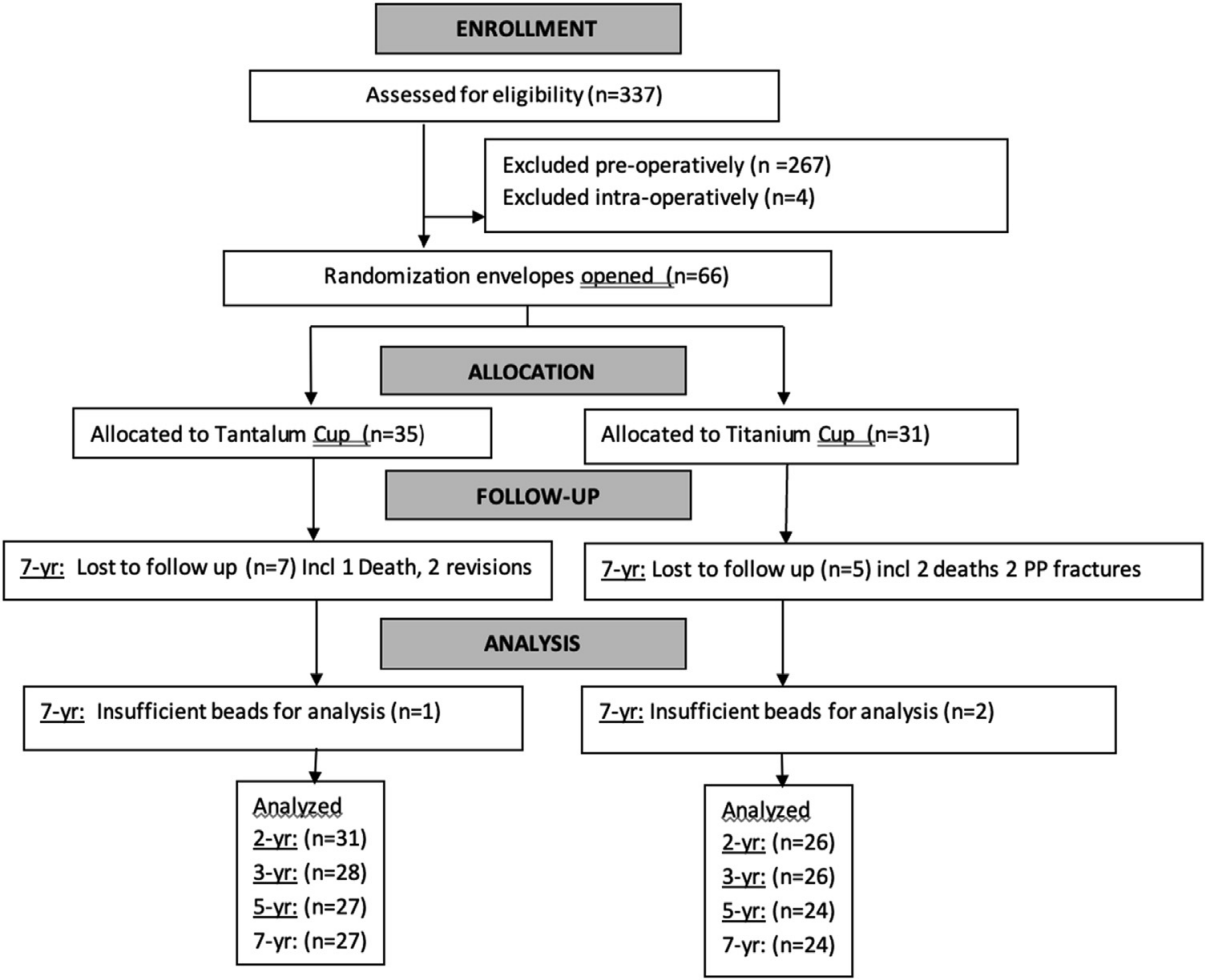
CONSORT flow diagram. CONSORT, Consolidated Standards of Reporting Trials; PP, Periprosthetic.

Patients were recruited between March 2007 and October 2013. The number of patients who were screened for eligibility, who were excluded pre-operatively or intra-operatively, and who were randomized and included in the analyses at 2, 3, 5, and 7 years are shown in Figure 1. Fifty-one (77.2%) of the 66 patients enrolled were included in the final 7-year analysis. Prior to 7-year follow-up, 2 tantalum components were revised due to recurrent dislocation and infection, respectively. Two additional patients with titanium components underwent open reduction internal fixation to treat femoral periprosthetic fracture. Three patients died (one tantalum and 2 titanium), 5 patients were lost to follow-up (four tantalum and one titanium). Three additional patients were excluded because RSA was not possible due to insufficient bead visualization (one tantalum and 2 titanium).

Patients randomized to a tantalum component were similar to those randomized to a titanium component with respect to age and body mass index. However, the proportion of men in the titanium cohort was greater than that in the tantalum cohort [12].

**Figure 2:**
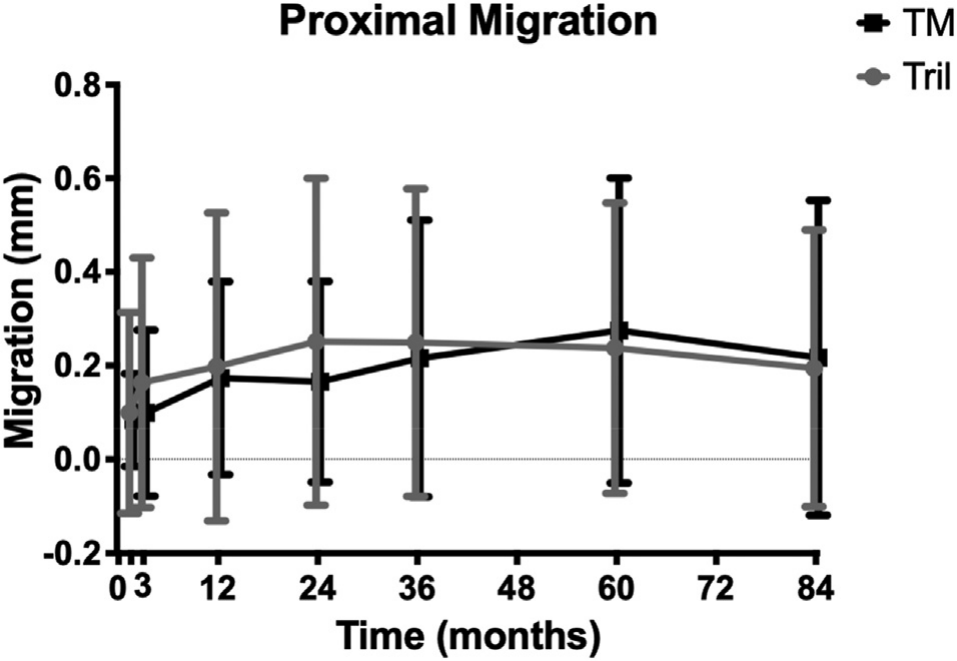
The mean proximal migration of each cohort at each RSA time interval. Error bars represent 95% confidence interval. TM, Tantalum; Tril, trilogy.

**Figure 3:**
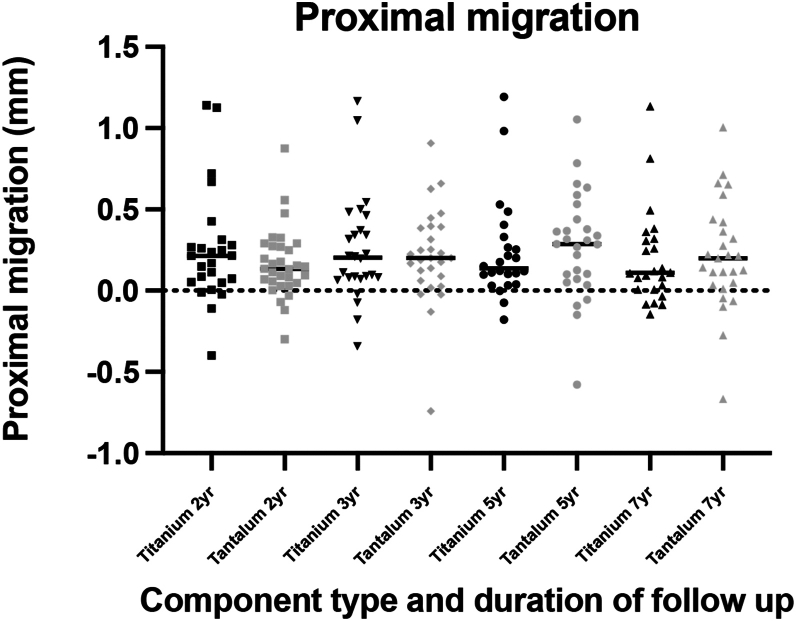
The individual proximal migration at 2, 5, and 7 years for each individual in the titanium and tantalum cohorts. The line is at mean.

The mean proximal migration was non-inferior between tantalum and titanium cohorts at 5 and 7 years (Fig. 2). At 5 years, the mean proximal migration for tantalum components was 0.28 mm (95% CI 0.15-0.40), and for titanium components, 0.24 mm (95% CI 0.10-0.37). At 7 years, the mean proximal migration of tantalum components was 0.22 mm (95% CI 0.08-0.35), and for titanium components, 0.19 mm (95% CI 0.07-0.32). There were no significant differences noted between cohorts for any other axis of translation or in trends of individual migraton (Fig. 3). However, there was significantly less change in inclination for tantalum components compared to titanium (*P* = .0481) (Table 1).

**Table 1:** Clinical outcomes, according to allocation to acetabular component type.

Harris Hip Score at follow-up	Tantalum	Titanium
Modified Harris Hip Score		
Pre-operative	35 (30-45)	36 (31-42)
2 y	91 (69-97)	90 (67-96)
5 y	89 (73-93)	83 (68-92)
7 y	92 (81-96)	81 (64-91)
Pain score		
Pre-operative	10 (10-10)	10 (10-20)
2 y	44 (44-44)	44 (40-44)
5 y	44 (44-44)	44 (40-44)
7 y	44 (44-44)	44 (40-44)

Median Modified Harris Hip and Pain Scores [20] were similar between cohorts both pre-operatively and at 7-year follow-up (Table 2).

**Table 2:** Translation and rotation at 7 years following THA, according to acetabular component type.

Axis	Tantalum (n = 27)		Titanium (n = 24)		Difference between means	95% Lower CI limit of difference	*P* value
	Mean	95% CI	Mean	95% CI			
Translation (mm)							
Medial (x-axis)	−0.08	−0.97 to 0.82	0.03	−1.85 to −0.04	−0.11	**−**1.06	.82
Proximal (y-axis)	0.22	0.08-0.35	0.19	0.07-0.32	0.02	−0.16	.80
Anterior (z-axis)	0.31	−0.39 to 1.01	0.06	−0.19 to 0.31	0.25	−0.48	.49
2D^a^	1.34	0.33-2.36	0.80	0.49-1.11	0.54	−0.51	.30
3D^b^	1.04	0.23-1.84	0.59	0.29-0.89	0.45	−0.40	.29
Rotation (degrees)							
Anterior tilt (x-axis)	0.49	−0.87 to 1.84	1.85	0.82-2.88	−1.36	−302	.11
Internal rotation (y-axis)	0.45	−0.91 to 1.82	1.86	0.40-3.3	−1.40	−3.34	.15
Sagittal rotation (z-axis)	−0.68	−1.97 to 0.62	−0.94	−1.85 to −0.05	−0.27	−1.30	.72

a Two-dimensional vectorial sum of migration.

b Three-dimensional vectorial sum of migration.

## Discussion

Tantalum acetabular components used in primary THA have been identified by recent registry studies to have an increased risk of revision [4,5]. Early component migration is a predictor of later loosening [7], but only one study has used sensitive RSA measurements to investigate the stability of tantalum components beyond 2 years and found continuing migration between 22 and 5 years [14]. When examining the broader literature on mid- to long-term acetabular component migration, there are only 13 RSA studies that report migration of any acetabular components at >5 years of follow-up [9].Our 7-year follow-up of patients enrolled into a RCT found the migration of tantalum components without ancillary fixation is non-inferior to the migration of titanium components with ancillary screw fixation. Equivalence of the stability achieved with tantalum components against titanium components, that have excellent clinical and survivorship performance [21,22], provides reassurance for their long-term survivorship.

Tantalum acetabular components have been used successfully in the revision settings to treat cases with poorer bone stock [23,24]. Tantalum metal provides a very porous surface with a higher coefficient of friction and inherently has a very different fixation to the fiber metal titanium components [3]. It is hypothesized that tantalum components may have been subsequently used in more complex primary THA cases which has inadvertently led to tantalum components being identified as an at-risk component in registry studies. There is a notable cost difference, with tantalum acetabular components accounting for a 27% price increase compared to titanium components. Our study's cohort of middle-aged patients with primary osteoarthritis treated at a large tertiary hospital would be a suitable cohort from which to draw comparisons for the performance of tantalum components in simple primary THA. Our RCT found low migration of both titanium and tantalum component cohorts. Due to the expected small increase in migration at 7 years, it is particularly reassuring that the tantalum components are shown to be non-inferior using the same threshold described in our 2-year paper of <0.21-mm change. A threshold of unacceptable mean cup migration of a cohort at 5- or 7-year follow-up is not described in the literature. Importantly, no individual component in the tantalum cohort migrated more than 1.29 mm, which has previously been identified as an unacceptable individual migration threshold associated with increased risk of revision of cemented acetabular components [25]. At this point in time, no individual migration thresholds exist for uncemented components following primary THA.

Table 3 summarizes the proximal migration of titanium and tantalum components in our current study compared to that of 2 previous RSA studies. Across all 3 RSA studies, no significance was noted at any timeframe, and future reports are required to determine if the ongoing migration of tantalum components is of concern. When comparing our 2-year results of tantalum components directly to those of Baad-Hansen et al. [13], similar low rates of proximal migration of tantalum acetabular components are observed [12,13]. Tantalum components in this cohort have continued to migrate proximally from 0.17 mm at 2 years [12] to 0.22 mm at 7 years and appears to have stabilized since the 5-year migration results of 0.28 mm. Ayers et al. [14] report the only migration to date at 5 years, showing an increase in tantalum acetabular components at all time frames except 3 years. Despite the increased migration of tantalum components of 0.21 mm compared with that of titanium at 0.05, it is acknowledged in their report that no statistical significance was found potentially due to 2 titanium components having greater than 2-mm migration increasing the standard error of the cohort. In our RCT, only 2 components migrated greater than 1 mm, with one from each of the titanium and tantalum cohort. However, the very low migration of titanium components reported by Ayers et al. [14] is not directly comparable to the “baseline scan” at 6 weeks, and our results show that a significant amount of migration has already occurred at this timepoint.

**Table 3:** Comparison of current results to RSA studies of proximal migration tantalum and titanium components.

Comparitive study	2 y			3 y			5 y			7 y		
	TM	TI	*P* value	TM	TI	*P* value	TM	TI	*P* value	TM	TI	*P* value
Baad-Hansen et al. [13]	0.08 (−0.22 to 0.38)	0.18 (−0.27 to 0.63)	.2	NR	NR	NR	NR	NR	NR	NR	NR	NR
Ayers et al. [14]	0.06 ± 0.04	0.05 ± 0.10	NR	0.03 ± 0.20	0.00 ± 0.07	NR	0.21 ± 0.05	0.05 ± 0.20	NR	NR	NR	NR
Current study cohort	0.17 (0.09-0.24)	0.19 (0.07-0.32)	.49	0.22 (0.10-0.33)	0.25 (0.12-0.38)	.69	0.28 (0.15-0.40)	0.24 (0.11-0.37)	.75	0.217 (0.08-0.35)	0.195 (0.07-0.032)	.80

NR, not reported; RSA, radiostereometric analysis: TM, tantalum; Ti, Titanium.

Baad-Hansen and current study mean with 95% confidence interval. Ayers median with standard error.

There is currently no threshold for continuing migration similar to what has been described in RSA studies of total knee arthroplasty implants [26]. As such, the significance of the continued migration seen in both our RCT and the 5-year results of Ayers et al. [14] is unknown. Our additional longer follow-up at 7 years confirmed component stabilization with no additional migration. In regard to the difference of z rotation or sagittal inclination between cohorts, the means −0.93 and 0.64 for tantalum and titanium represent less than 1° change in inclination and is very unlikely to entail a significant clinical change.

Our RCT found that the pre-operative Harris hip, pain, and activity scores were similar between cohorts. Both cohorts showed a good improvement in their post-operative clinical outcomes. This finding was to be expected, and other similar RCTs have shown comparable clinical outcomes [13,14]. Overall, the low migration results demonstrate non-inferiority of tantalum components and contribute to paucity of knowledge surrounding early- and mid-term migration of tantalum acetabular components and are strengthened by accurate RSA measurements of migration with good precision. One limitation of our study is that 15 of the 66 patients who were originally randomized were not available for analysis at 7-year follow-up after THA. However, the variation in the data is so small that the titanium cohort was deemed to be of sufficient size. An additional limitation is only one observer performed the RSA examination; a double examination may have reduced the risk of bias. It is also acknowledged that our study did not include a cohort of titanium acetabular components without screw fixation. However, migration of titanium components with and without screw fixation has been extensively studied previously and shown to result in no difference in migration [[27], [28], [29]].

## Conclusions

This RSA study confirms the continued mid-term stability of a highly porous coated solid-backed tantalum acetabular component with no ancillary screw fixation and titanium acetabular components with ancillary screw fixation in relatively young patients undergoing primary THA. The non-inferiority of tantalum component migration provides some early reassurance about the long-term survival of primary acetabular components; however, further reports are required to determine the long-term tantalum component stability.

## Funding

This study was funded primarily by a Project Grant from the 10.13039/501100000925National Health and Medical Research Council of Australia (#1028392). Some initial funding was also received from Zimmer Biomet and a Research Development Award from the Faculty of Health Sciences at the 10.13039/501100001786University of Adelaide. Funds were used for salary support and for research-related activities. Funding sources had no role in study design, data collection and analysis, decision to publish, or manuscript preparation.

## Conflicts of interest

Subsequently author SC held an EMCR Research Fellowship from The Hospital Research Foundation Group during the period of this study. LBS is in the speakers bureau of/gave paid presentations for AO recon and receives research grant from Zimmer Biomet. DWH and SAC receives research support from Zimmer Biomet.

For full disclosure statements refer to https://doi.org/10.1016/j.artd.2025.101709.

## CRediT authorship contribution statement

**Thomas S. Robertson:** Writing – review & editing, Writing – original draft, Validation, Supervision, Investigation, Funding acquisition, Formal analysis, Data curation. **Lucian B. Solomon:** Writing – review & editing, Writing – original draft, Validation, Data curation, Conceptualization. **Donald W. Howie:** Writing – review & editing, Writing – original draft, Formal analysis, Conceptualization. **Oksana T. Holubowycz:** Writing – review & editing, Writing – original draft, Supervision, Formal analysis, Conceptualization. **Chan Hee Cho:** Writing – review & editing, Writing – original draft, Validation, Software, Formal analysis, Data curation. **Stuart A. Callary:** Writing – review & editing, Writing – original draft, Supervision, Project administration, Methodology, Investigation, Conceptualization.

## Figures and Tables

**Appendix 1:** Numbers of patients excluded pre-operatively according to exclusion criteria.

Exclusion criterion	No. excluded^a^
Too young (<40 y old)	20
Too old (>64 y old)	125
Planned simultaneous bilateral total hip arthroplasty	0
Opposite hip already in this trial	14
Diagnosis other than osteoarthritis	30
Planned prosthesis not CPT 12/14	0
Planned prosthesis not a 28-mm non-skirted head	3
Planned prosthesis not Trilogy or TM acetabular system with Longevity polyethylene 10° elevated rim liner	0
Planned approach other than posterior	0
Charnley class C	15
Inflammatory arthritis	1
Previous infection in this hip	2
Crowe 2 DDH or worse dysplasia	4
Acetabular deficiency—ie, >2 cm superior loss acetabular dome or >1.5 cm acetabular protrusion or wall deficiency >1/2 wall	8
Current or previous primary or metastatic tumor involving this hip	0
Current or previous pelvic irradiation	2
Current cytotoxic treatment/anti-metabolites/chemotherapeutic agent	0
Current steroid treatment	2
Other causes of severe osteopenia or potentially delayed bone ingrowth	0
Currently on bisphosphonates	1
Unable to provide informed consent (insufficient English or cognitive disorder [eg, dementia] or psychiatric illness)	6
Unable to complete follow-up (life expectancy <2 y or insufficient English for questionnaires or unable to return easily)	26
Total	259

TM, tantalum.

a Patients were excluded in a hierarchical manner, with only the first listed relevant exclusion criterion being recorded.

## Appendix A Supplementary data

Conflict of Interest Statement for Howie

Conflict of Interest Statement for Holubowycz

Conflict of Interest Statement for Callary

Conflict of Interest Statement for Cho

Conflict of Interest Statement for Robertson

Conflict of Interest Statement for Solomon
