# Supplementary material for: Highly Porous Tantalum Acetabular Components Without Ancillary Screws Are Non-inferior at 7 Years When Compared With Titanium Components With Ancillary Screw Fixation: A Randomized Controlled Trial
Source: Arthroplast Today. 2025 May 17;33:101709. doi: 10.1016/j.artd.2025.101709 (PMC12145538; doi:10.1016/j.artd.2025.101709)
Supplement: Conflict of Interest Statement for Howie [file mmc1.pdf]

INDIVIDUAL CONFLICT OF INTEREST STATEMENT

American Association of Hip and Knee Surgeons

(Adopted from the American Academy of Orthopaedic Surgeons disclosure statement)

The following form **must be filled out completely and submitted by each author (example, 6 authors, 6 forms). All items require a response. If there is no relevant disclosure for a given item, enter "*None.*"**

Manuscript Title: Highly porous tantalum acetabular components without ancillary screws are non-inferior at seven years when compared with titanium components with ancillary screw fixation: a randomized controlled trial

1. Royalties from a company or supplier (The following conflicts were disclosed)
   Nil

2. Speakers bureau/paid presentations for a company or supplier (The following conflicts were disclosed)
   Nil

3A. Paid employee for a company or supplier (The following conflicts were disclosed)
   Nil

3B. Paid consultant for a company or supplier (The following conflicts were disclosed)
   Nil

3C. Unpaid consultants for a company or supplier (The following conflicts were disclosed)
   Nil

4. Stock or stock options in a company or supplier (The following conflicts were disclosed)
   Nil

5. Research support from a company or supplier as a Principal Investigator (The following conflicts were disclosed)
   ZimmerBiomet

6. Other financial or material support from a company or supplier (The following conflicts were disclosed)
   Nil

7. Royalties, financial or material support from publishers (The following conflicts were disclosed)

   Nil

8. Medical/Orthopaedic publications editorial/governing board (The following conflicts were disclosed)
   Nil

9. Board member/committee appointments for a society (The following conflicts were disclosed)
   Nil

Each author must sign AND print or type his/her name, date and submit a separate form
In addition, one BLINDED Conflict of Interest form (no author names used) should be submitted per manuscript with all author disclosures.

Donald Howie	[signature]	5-3-2024
Author Name:	Author Signature	Date
